# Bug Wars: Artificial Intelligence Strikes Back in Sepsis Management

**DOI:** 10.3390/diagnostics15151890

**Published:** 2025-07-28

**Authors:** Georgios I. Barkas, Ilias E. Dimeas, Ourania S. Kotsiou

**Affiliations:** 1 Laboratory of Human Pathophysiology, Department of Nursing, School of Health Sciences, University of Thessaly, 41500 Larissa, Greece; gbarkas-m@uth.gr; 2 Department of Respiratory Medicine, Faculty of Medicine, School of Health Sciences, University of Thessaly, 41110 Larissa, Greece; dimel13@hotmail.com

**Keywords:** artificial intelligence, machine learning, prediction, sepsis

## Abstract

Sepsis remains a leading global cause of mortality, with delayed recognition and empirical antibiotic overuse fueling poor outcomes and rising antimicrobial resistance. This systematic scoping review evaluates the current landscape of artificial intelligence (AI) and machine learning (ML) applications in sepsis care, focusing on early detection, personalized antibiotic management, and resistance forecasting. Literature from 2019 to 2025 was systematically reviewed following PRISMA-ScR guidelines. A total of 129 full-text articles were analyzed, with study quality assessed via the JBI and QUADAS-2 tools. AI-based models demonstrated robust predictive performance for early sepsis detection (AUROC 0.68–0.99), antibiotic stewardship, and resistance prediction. Notable tools, such as InSight and KI.SEP, leveraged multimodal clinical and biomarker data to provide actionable, real-time support and facilitate timely interventions. AI-driven platforms showed potential to reduce inappropriate antibiotic use and nephrotoxicity while optimizing outcomes. However, most models are limited by single-center data, variable interpretability, and insufficient real-world validation. Key challenges remain regarding data integration, algorithmic bias, and ethical implementation. Future research should prioritize multicenter validation, seamless integration with clinical workflows, and robust ethical frameworks to ensure safe, equitable, and effective adoption. AI and ML hold significant promise to transform sepsis management, but their clinical impact depends on transparent, validated, and user-centered deployment.

## 1. Introduction

Sepsis represents a critical global health concern, accounting for an estimated 11 million deaths annually, which translates to roughly 1 in 5 deaths worldwide [1,2]. In high-income countries, such as the United States, sepsis results in over 2 million hospitalizations and approximately 350,000 deaths each year, with healthcare costs surpassing USD 52 billion [3,4]. The burden is especially pronounced among vulnerable populations, including neonates, the elderly, cancer patients, and the immunocompromised, who experience disproportionately higher mortality rates [5,6]. Despite international awareness campaigns and updated treatment guidelines, sepsis continues to challenge healthcare systems due to its high incidence, rapid progression, and heterogenous clinical presentations.

Early recognition of sepsis is vital, as delays in diagnosis and therapy initiation are directly correlated with increased mortality. Evidence indicates that mortality rises by up to 8% for each hour of delay in administering effective antimicrobial therapy [7,8,9,10]. Diagnosis is currently guided by clinical criteria such as the Sequential Organ Failure Assessment (SOFA) and the quick SOFA (qSOFA) scores [11,12,13]. However, these tools, while useful for prognosis, often detect sepsis after organ dysfunction has already begun, limiting opportunities for timely intervention. Standard treatment approaches—including fluid resuscitation and empiric antibiotic administration—can be lifesaving, yet they are often implemented late in the disease’s course and lack personalization [14,15,16].

A compounding challenge in sepsis management is antimicrobial resistance (AMR). Globally, AMR contributes to over 1 million deaths annually and is a significant factor in poor sepsis outcomes, especially in Internal Care Unit (ICU) settings [17,18]. In practice, empiric regimens, such as vancomycin with piperacillin–tazobactam, are frequently initiated before pathogen identification, yet 30–50% of these therapies are inappropriate due to microbial resistance or misdiagnosis [19,20]. Conventional culture-based testing requires 48–72 h, leaving clinicians reliant on broad-spectrum antibiotics that may be ineffective, toxic, or both. This not only worsens outcomes but also drives the cycle of resistance and healthcare-associated infections [19,20].

In this context, artificial intelligence (AI) has emerged as a promising solution to bridge diagnostic and therapeutic gaps in sepsis care. AI-powered systems can analyze vast amounts of clinical, microbiological, and pharmacological data in real time, offering predictive insights that surpass traditional tools. For example, machine learning (ML) algorithms have shown the ability to detect sepsis onset 4 to 12 h before clinical recognition, and AI-based decision support systems can recommend targeted antibiotic regimens, reduce adverse drug events, and support timely de-escalation strategies [19,21,22,23,24]. Integrating biomarkers, genomic data, and electronic medical records (EMRs), these tools offer a precision medicine approach to what has traditionally been a reactive and generalized treatment process.

Despite the encouraging progress, several barriers prevent widespread clinical adoption of AI in sepsis care. These include limited external validation of models, lack of interpretability, incomplete integration into electronic health record systems, and resistance from clinicians due to trust and workflow concerns [25,26]. Additionally, existing models often lack generalizability across diverse patient populations and care settings. This review aims to explore the current landscape of AI applications in sepsis, with a focus on their role in early detection, antibiotic guidance, and resistance prediction. By highlighting validated approaches and identifying research and implementation gaps, we seek to provide a comprehensive overview of how AI can transform sepsis management and antimicrobial stewardship.

## 2. Methods

### 2.1. Study Design and Eligibility Criteria

The present review was conducted systematically in accordance with the PRISMA-ScR 2020 guidelines. Inclusion criteria encompassed original studies involving adults aged ≥16 years with suspected or confirmed sepsis and investigating artificial intelligence (AI) or machine learning (ML) models targeting sepsis detection, treatment, or antimicrobial resistance forecasting. Eligible studies included prospective and retrospective analyses, randomized controlled trials (RCTs), and quasi-experimental designs, published in English between January 2019 and April 2025.

### 2.2. Information Sources and Search Strategy

Literature searches were systematically conducted using the PubMed database (https://pubmed.ncbi.nlm.nih.gov, accessed 25 April 2025). Search terms included “Artificial Intelligence AND sepsis” and “Machine Learning AND sepsis,” and the results were filtered to include only English-language articles published within the last five years (2019–2025).

### 2.3. Study Selection Process

**Figure 1:**
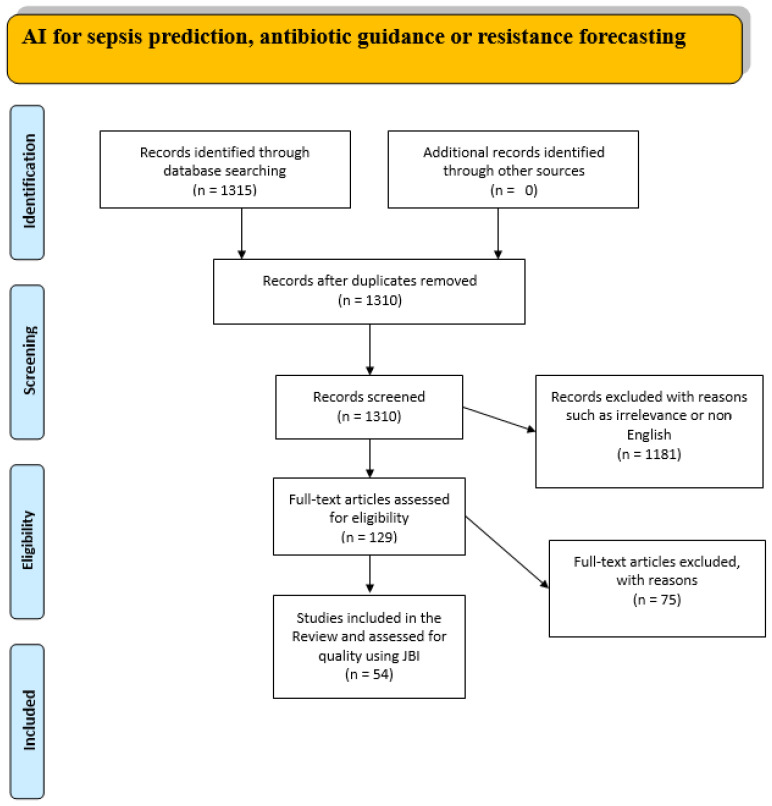
The flowchart of the study.

The initial search yielded 1315 records. Titles and abstracts were independently screened by two reviewers (GB, ID). Disagreements were resolved through consultation with a third reviewer (OSK). Ultimately, 129 articles underwent full-text review. Exclusion criteria were applied, removing articles not published in English, older than five years, or irrelevant to AI or ML applications in sepsis. Additionally, references from selected articles were manually checked to identify further relevant studies. Extracted data included study setting, sample size, type of AI methodology employed, data source, external validation status, and clinical outcomes. A narrative synthesis was conducted, categorizing findings according to the primary AI function: sepsis prediction, antibiotic guidance, or resistance forecasting. The literature identification, screening, eligibility, and final inclusion processes are illustrated in Figure 1, depicting the systematic approach taken to select relevant studies for review.

### 2.4. Quality Assessment

Additionally, quality assessment of the 54 included studies was performed using the Joanna Briggs Institute (JBI) critical appraisal tool, appropriate to each study design. The proportion of fulfilled JBI criteria per study ranged from 28% to 100%, with a median score of approximately 85%. Twenty-four studies achieved a score of 90% or above (“excellent” quality), ten studies were classified as “good” (80–89%), ten studies as “moderate” (70–79%), and seven studies as “fair” (60–69%). Only three studies were categorized as “low quality” (<60%). Most studies demonstrated robust methodological quality; however, recurring limitations included incomplete reporting of confounding factors, lack of clarity in participant selection, and limited follow-up in some cases. A detailed, item-level summary of the JBI appraisal is provided in Supplementary Table S1.

### 2.5. Risk of Bias Assessment

**Figure 2:**
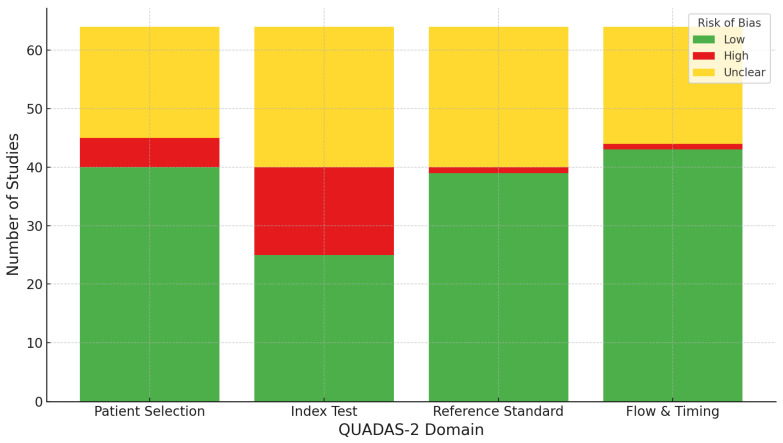
Risk of bias judgments by QUADAS-2 domain. Note: A stacked bar chart heatmap visualizing the aggregate risk of bias judgments by QUADAS-2 domain. Each bar represents a domain, with colors indicating the number of studies rated as low (green), high (red), or unclear (yellow) risk of bias for each.

Furthermore, an aggregate risk of bias assessment using the QUADAS-2 tool is presented in Supplementary Table S1. Figure 2 provides a visual summary of the risk of bias assessment across four QUADAS-2 domains (Patient Selection, Index Test, Reference Standard, Flow and Timing) for the 47 included primary research studies, as QUADAS-2 is specifically designed to assess the risk of bias in primary diagnostic accuracy studies, not reviews, meta-analyses, or secondary literature. The majority of studies were rated as having low risk of bias (green), particularly in the “Flow & Timing” and “Reference Standard” domains. However, a considerable proportion of studies were judged as having unclear risk (yellow) across all domains, highlighting the frequent lack of sufficient methodological detail in reporting. Notably, the “Index Test” domain exhibited the highest number of studies with high risk of bias (red), emphasizing methodological concerns related to test conduct or interpretation. Overall, while the aggregate assessment suggests acceptable methodological quality in most domains, the prevalence of unclear and high-risk judgments—especially in the Index Test—indicates persistent issues that could impact the validity and generalizability of the findings. These results underline the need for improved reporting standards and methodological rigor in future diagnostic studies.

## 3. Results

### 3.1. Sepsis Detection in Daily Clinical Practice

Early and accurate detection of sepsis is crucial to improving patient outcomes. Currently, several clinical scoring systems and biomarkers are used in practice to identify sepsis and assess its severity. Among these, the SOFA score and the qSOFA score are widely utilized.

The SOFA score evaluates six organ systems—respiratory, cardiovascular, hepatic, coagulation, renal, and neurological—assigning points based on the degree of dysfunction. It has been shown to have high prognostic accuracy for in-hospital mortality among critically ill patients with suspected infection [11,12,13]. In contrast, the qSOFA score, which includes three variables (altered mental status, respiratory rate ≥ 22/min, and systolic blood pressure ≤ 100 mmHg), is intended for rapid bedside assessment. However, studies have demonstrated that qSOFA has lower sensitivity than SOFA, particularly in non-ICU settings, and may miss early sepsis cases [11,12].

In addition to clinical scores, biomarkers, such as procalcitonin (PCT) and C-reactive protein (CRP), are frequently used to support sepsis diagnosis and monitor disease progression. Procalcitonin is a precursor of the hormone calcitonin, which increases in response to bacterial infection and systemic inflammation. It is particularly useful for distinguishing bacterial from viral infections and has been incorporated into antibiotic stewardship protocols to guide initiation and discontinuation of therapy [27,28,29]. Elevated PCT levels correlate with sepsis severity and have been associated with increased mortality in ICU patients [27,28].

Similarly, CRP is a nonspecific acute-phase protein that rises during systemic inflammation. Although widely available and inexpensive, CRP lacks specificity and can be elevated in a range of non-infectious conditions, limiting its standalone diagnostic value in sepsis [29,30]. However, when interpreted in conjunction with clinical findings and other markers, CRP remains a helpful component in the early identification and monitoring of septic patients.

Overall, while scoring systems like SOFA provide a standardized approach to quantifying organ dysfunction, and biomarkers, such as PCT and CRP, offer supportive diagnostic information, limitations in sensitivity, specificity, and real-time responsiveness persist. These gaps underscore the need for advanced, integrative approaches, such as those offered by artificial intelligence models, to improve the timeliness and accuracy of sepsis detection.

### 3.2. AI in Sepsis Prediction

AI, particularly ML and deep learning, has shown great promise in sepsis prediction by enabling earlier and more accurate identification of at-risk patients compared to conventional scoring systems [31]. Over the past decade, several AI models have emerged, demonstrating varying levels of accuracy, interpretability, and real-world applicability. Reported Area Under the Receiver Operating Characteristic Curve (AUROC) values range from 0.68 to 0.99, with predictive windows extending up to 12 h before onset, surpassing traditional systems in both sensitivity and specificity. Among the most extensively studied are DeepAISE, InSight, and the Nemati model [32,33,34,35,36].

Multiple studies have shown that AI-based models achieve superior real-time prediction of sepsis risk, often providing interpretable outputs that assist in clinical understanding and decision making, especially in the ICU [32].

For example, Li et al. developed and validated the Time-phased machine learning model for Sepsis Prediction (TASP) to provide real-time sepsis onset prediction in critical care [33]. Using retrospective cohort data from three U.S. hospitals, they constructed 312 hourly features for each ICU stay and trained a LightGBM classifier using five-fold cross-validation. A novel “time-phased” strategy, employing three different cutoffs with length of stay, converted predicted likelihoods into actionable binary predictions, improving both accuracy and clinical interpretability. The TASP model achieved an AUROC of 0.845 and demonstrated substantial utility in both internal and test validation sets, including a utility score of 0.430 on the internal validation set and 0.354 on the test set [33].

DeepAISE (Deep Artificial Intelligence Sepsis Expert) is a real-time deep learning model that utilizes continuous electronic health record (EHR) data to predict the onset of sepsis [34]. It was trained on extensive clinical datasets and is notable for its time-phased architecture, which models patient physiology over time rather than relying on static snapshots. In a multicenter evaluation, DeepAISE demonstrated high predictive accuracy, with AUROC values exceeding 0.90 for early sepsis detection in ICU settings. A key strength of DeepAISE is its adaptability to heterogeneous data streams, including vital signs, lab results, and interventions, making it robust across patient populations [34].

Developed by Desautels et al., InSight is another leading ML-based tool for sepsis prediction. It is designed to work with minimal data inputs—often just six vital signs—making it highly adaptable, especially in low-resource environments [35]. InSight achieved an AUROC of 0.880 (SD 0.006) and an Area Under the Precision–Recall curve (APR) of 0.595 (SD 0.016) at the time of sepsis onset. These performance metrics were superior to those of SIRS, qSOFA, and MEWS when computed concurrently, as well as SAPS II and SOFA computed at admission. The AUROC for InSight at 4 h preceding sepsis onset was 0.74 (SD 0.010), while the APR was approximately 0.27. While the AUROC remains competitive, the APR shows a decrease as the prediction time moves further from the onset of sepsis. Specifically, at 4 h prior to sepsis onset, InSight’s performance in terms of AUROC was comparable to or better than other methods, but its APR was lower. However, even with approximately 60% of input data deleted at random, InSight maintained superior classification performance compared to these other scores [35]. Its relatively light computational footprint allows for integration into bedside monitoring systems, and it has been validated in both retrospective and real-time environments. However, like most early models, its transparency and interpretability have been points of critique.

A prospective observational study aimed to externally validate the VitalCare-SEPsis Score (VC-SEPS), a deep-learning-based AI algorithm for early sepsis prediction and risk stratification in hospitalized patients using real-world electronic medical record (EMR) data [36]. The intervention involved deploying VC-SEPS to generate dynamic risk scores hours before sepsis onset, with comparisons to traditional scoring tools (SOFA and qSOFA) in adult ward patients at a South Korean hospital. Results showed VC-SEPS achieved an AUROC of 0.880, higher than SOFA and qSOFA, and an accuracy of 87.35% versus 62.35% (SOFA) and 62.96% (qSOFA). VC-SEPS predicted sepsis an average of 68.05 min before clinical diagnosis, delivered a high NPV (0.997), and successfully stratified patients into risk groups—those with higher initial VC-SEPS scores had significantly greater sepsis risk, even after adjustment for confounders. The algorithm also outperformed traditional methods in both community- and hospital-onset sepsis subgroups and consistently provided more timely and accurate early warnings, supporting improved clinical decision making and resource allocation [36].

Zhao et al. focused on patients with sepsis-associated acute kidney injury (SA-AKI), successfully identifying 12 critical risk factors for early prediction. In their study, they developed a web-based application incorporating four different machine learning models and applied SHapley Additive exPlanations (SHAP) techniques to enhance model interpretability [37]. Notably, their analysis identified atrial fibrillation as a novel predictor of AKI and highlighted body weight as the most influential factor, as revealed through SHAP analysis. Among the tested models, the gradient boosting machine achieved the highest predictive performance, with an AUC of 0.794, an accuracy of 78.3%, sensitivity of 94.2%, specificity of 32.1%, and a Brier score of 0.150 (the lowest, indicating the best calibration) in the development cohort. These results demonstrate that the models can reliably predict SA-AKI within the critical 48 h window. Furthermore, the web-based nature of the tool allows for broad clinical implementation and facilitates the selection of optimal risk thresholds for clinical use [37].

Using complete blood count parameters and monocyte distribution width (MDW), the researchers sought to overcome the poor sensitivity and positive predictive value associated with individual sepsis biomarkers [38]. They developed and externally validated machine learning models for early sepsis detection, training five different algorithms on data from 5344 patients across six Italian hospital cohorts. The resulting models demonstrated excellent performance, with AUC values ranging from 0.91 to 0.98 during internal validation and robust generalizability in external validation datasets (AUC 0.75–0.95). Across hospital settings, the models achieved sensitivities of 58–95%, specificities of 78–97%, positive predictive values (PPV) of 28–72%, and consistently high negative predictive values (NPV) of 96–100%. Among the algorithms tested, the extreme gradient boosting model was the top performer, achieving an AUC of 0.98 and an average PPV of 0.83. This significantly outperformed both traditional biomarker thresholds and existing state-of-the-art approaches. Moreover, the implementation of cautious classification strategies further improved both sensitivity and PPV, with minimal impact on overall coverage [38].

An important tool in the field is the NAVOY Sepsis algorithm, which leverages four hours of routinely collected clinical data—including vital parameters, blood gas values, and laboratory results—for early sepsis prediction [39]. In its pivotal validation study, which represents the largest randomized clinical trial conducted to date for a machine learning sepsis prediction algorithm and the first to clinically validate such a tool against Sepsis-3 criteria, NAVOY Sepsis demonstrated the ability to predict sepsis development up to three hours before onset with high performance in a prospective ICU setting. This robust validation established NAVOY Sepsis as a clinically viable solution for early sepsis detection. The algorithm achieved an accuracy of 0.79, correctly predicting sepsis status in 79% of cases. It demonstrated high sensitivity (0.80), accurately identifying 80% of patients who would develop sepsis, while maintaining good specificity (0.78), correctly excluding 78% of patients who would not develop sepsis. The AUROC was 0.80, reflecting good discriminative ability to distinguish between septic and non-septic patients. When the algorithm predicted sepsis, the positive predictive value (PPV) was 0.53, indicating a 53% likelihood that a positive prediction corresponded to an actual sepsis case, while the negative predictive value (NPV) was 0.93, meaning that when sepsis was not predicted, there was a 93% probability the patient truly would not become septic [39].

The study by Shi et al. aimed to develop an ML-based prediction model for early identification of high-risk sepsis patients in the ICU by analyzing clinical data from the first 24 h of admission [40]. The researchers extracted 31 demographic and clinical features from EHR to train and validate various ML algorithms, using both US (MIMIC-IV) and Chinese patient data to ensure cross-cultural applicability. The study evaluated model performance using metrics like test accuracy scores and the Area Under the Receiver Operating Characteristic Curve (AUC), with machine learning models—especially Gradient Boosting Machine—substantially outperforming traditional linear regression (GBM test score 0.78, AUC > 0.8, compared to linear regression score of 0.25) and maintaining robust results in external validation (scores 0.63–0.77). To address the issue of interpretability, SHAP (SHapley Additive exPlanations) visualizations were employed, which allowed for both global insight into the importance of clinical features and individualized patient-level risk explanations. The results demonstrated that this ML approach could accurately and consistently predict sepsis mortality risk across different healthcare environments, providing a valuable tool for supporting early intervention and improving patient outcomes [40].

Nemati et al. developed an Artificial Intelligence Sepsis Expert (AISE) algorithm using a modified Weibull-Cox proportional hazards model that combines EMR data with high-resolution vital signs to predict sepsis onset 4–12 hours in advance [41]. The model incorporates clinical trajectories from EHRs, allowing it to learn temporal dependencies in patient data. One distinguishing feature is its interpretable output, which highlights which clinical features influenced each prediction. This is essential for clinical trust and adoption. The Nemati model demonstrated AUROC values between 0.83 and 0.85 and specificity and accuracy values between 0.63 and 0.67, predicting sepsis as early as 12 h before onset. Its strengths include a high level of personalization and the capacity for integration into real-time ICU monitoring systems. Table 1 provides a structured comparison of key models, detailing their algorithmic approaches (e.g., random forest, deep neural networks, ensemble learning), the characteristics and sources of their training data (single-center vs. multicenter, prospective vs. retrospective), and their validation strategies. This expanded comparison allows for a more nuanced understanding of the advantages and limitations inherent in each approach [41].

In summary, models like DeepAISE [34] and the Nemati RNN [41] utilize multimodal, time-phased EHR data to achieve high AUROC values (>0.90) and extended prediction windows, while others like InSight [35] perform robustly using only basic vital signs, supporting wider clinical use.

The NAVOY Sepsis algorithm stands out for its large, prospective validation (AUROC 0.80, sensitivity 0.80, specificity 0.78), and the VC-SEPS model demonstrated superior accuracy and early warnings compared to SOFA and qSOFA in real-world hospital settings. Incorporation of laboratory and genomic data, as seen in Zhao et al. [37] and multicenter Italian cohorts, further boosts predictive power and interpretability (AUCs up to 0.98, high NPV).

Model performance generally exceeds traditional risk scores, but generalizability and workflow integration remain challenges. While advanced deep learning models offer high accuracy, they often lack transparency, whereas simpler ML models, such as InSight, enable broader deployment but may be less sensitive to complex patterns. The use of explainable AI tools, like SHAP values, though still limited, enhances clinical trust (e.g., Shi, Zhao).

Beyond individual models, integrated approaches that combine biomarkers (e.g., procalcitonin, IL-6), genomic testing, and metabolomic profiling with EMR data have shown significant promise [44]. Studies report improvements in predictive accuracy when combining clinical data with biomarkers, raising AUROC values from 0.75 to 0.81 [41,42,45,46], and indicate that such multimodal frameworks may reduce in-hospital mortality from 21.3% to 8.96% [46,47]. Pathogen-specific biomarkers have been used in conjunction with ML in septic patients, and multicenter studies focused on simple ABG variables to predict sepsis and help physicians with decisions have had positive results [48,49]

In-hospital mortality in ICU patients with sepsis has also been studied, and prediction models have been created for both sepsis prediction as well as patient risk and patient susceptibility [50,51,52]. In an AI application developed and validated to assess trauma patient susceptibility to sepsis, an ML-based Sepsis Risk Index (SRI) was created and validated based on a 0-100 scale that reflects a patient’s risk of acquiring sepsis or septic shock [51]. The SRI demonstrated significant early warning capacity, successfully predicting sepsis diagnosis in 60.6% of patients before clinical documentation while also achieving an AUC of 0.82 for sepsis diagnosis and of 0.84 for septic shock. Unlike many previous studies, this research validated the model across multiple ICU’s using the elCU database, demonstrating more comprehensive and universal applicability. Meanwhile, a recent multicenter study specifically focused on major trauma patients in the ICU, and Sun et al. [51] developed and validated an AI tool using six ML models. This model used 12 predictors, including clinical and laboratory variables, and demonstrated strong performance. For internal validation, its AUC was 0.913, and for external validation, 0.886. The model was implemented as a free web-based application to help clinicians rapidly identify high-risk trauma patients and enable early interventions, with comprehensive evaluation across multiple metrics confirming its reliability and potential clinical utility [51].

Additional ML-driven early warning systems are under active development. These leverage continuous monitoring data, lab results, and vital signs to provide real-time alerts [53,54,55]. For instance, clinico-metabolomic models have improved mortality prediction compared to conventional tools [56,57,58], and rapid genomic diagnostics now enable faster pathogen identification, facilitating tailored interventions [59,60,61,62]. While research approaches also focus on comparing different ML algorithms to identify the most effective approach for this critical clinical prediction task, further showing ML’s future impact in everyday practice.

Despite these advancements, widespread adoption of AI in sepsis care is hindered by limited generalizability, data interoperability issues, and clinician skepticism stemming from a lack of interpretability and transparency [20,25,45]. Future research should prioritize multicenter validation, user-friendly design, and seamless integration with clinical workflows to realize the full potential of AI in sepsis management.

### 3.3. Limitations in Current Antibiotic Management of Sepsis

Traditional antibiotic management in sepsis emphasizes the rapid initiation of broad-spectrum empiric therapy, tailored to the presumed source of infection, patient-specific risk factors, and local resistance patterns [30]. Timely administration is critical. The 2016 Surviving Sepsis Campaign (SSC) recommends initiating antibiotics within one hour of recognition, a target supported by studies indicating a 7.6% increase in mortality for every hour of delay [63,64,65,66]. While early administration is widely accepted, strict adherence to the one-hour benchmark is debated. In non-shock cases, clinical judgment is encouraged to avoid unnecessary antibiotic overuse [67]. A 2025 study reported that although 95% of ICU physicians prioritize prompt antibiotic initiation, many face difficulties balancing urgency with antimicrobial stewardship (AS) principles [68].

Empiric regimens often include carbapenems (e.g., meropenem) or beta-lactam/beta-lactamase inhibitors (e.g., piperacillin–tazobactam) for Gram-negative coverage and vancomycin or linezolid for suspected methicillin-resistant *Staphylococcus aureus* (MRSA) [67,68]. In patients with suspected multidrug-resistant (MDR) infections or those at high risk, combination therapies, such as carbapenems with tigecycline or colistin, are frequently employed [68,69]. For septic shock, dual Gram-negative coverage (e.g., β-lactam plus aminoglycoside or fluoroquinolone) is recommended to improve empiric adequacy, as endorsed by the Infectious Diseases Society of America (IDSA) [67].

A major limitation of current practice lies in the dependence on conventional diagnostic tools, such as blood cultures and antimicrobial susceptibility testing (AST), which typically require 48–72 h for results [69]. This delay compels clinicians to initiate broad-spectrum antibiotics empirically, contributing to overtreatment. For instance, vancomycin is frequently overused, with up to 40% of courses unnecessarily prolonged, increasing the risk of nephrotoxicity, Clostridium difficile infection, and other adverse outcomes [67,69].

AMR further complicates empiric treatment. Resistance surveillance systems are often static and fail to reflect emerging or local resistance trends. For example, 83% of extended-spectrum beta-lactamase (ESBL)-producing *Escherichia coli* exhibit concurrent fluoroquinolone resistance, limiting therapeutic options [70]. Surveillance gaps also lead to underreporting of community-acquired MDR organisms, such as Panton-Valentine leukocidin (PVL)-producing MRSA clones [71]. Consequently, inappropriate antibiotic selection becomes more likely, fueling resistance and clinical failures [72].

Unnecessary broad-spectrum exposure is widespread. Studies show that over 50% of anti-MRSA and approximately 49% of antipseudomonal β-lactam use occurs in patients without confirmed infections by the targeted pathogens [73,74]. This misuse is associated with significant toxicity risks, as vancomycin-related nephrotoxicity ranges from 14% to 28%, and carbapenem overuse increases the likelihood of *Clostridium difficile* infection by 3.2-fold [75,76,77,78]. Despite rising awareness, broad-spectrum prescribing increased from 63% to 66.7% between 2017 and 2021, potentially driven by diagnostic delays and reliance on outdated resistance assumptions [71,73].

Delayed antibiotic de-escalation—where empiric broad-spectrum therapy is continued despite the availability of culture data—is another persistent challenge. Although AS programs promote narrowing therapy based on microbiological findings, implementation remains inconsistent [71]. Biomarkers, such as PCT, have demonstrated efficacy in guiding early cessation, reducing antibiotic duration by up to 35%, yet only 22% of emergency departments (EDs) utilize PCT protocols effectively [28,29,68]. Similarly, essential sepsis indicators, such as blood lactate, are often delayed, with more than 55% of cases showing measurement times exceeding one hour post-admission, despite their prognostic significance [67,79].

Diagnostic delays are strongly associated with worse outcomes. A recent meta-analysis showed that each hour of antibiotic delay increases in-hospital mortality (OR = 1.041, 95% CI: 1.021–1.062) [80]. Legal case reviews reveal that misdiagnosed sepsis patients had a 49% mortality rate, with many undergoing multiple outpatient visits before hospitalization [81]. Delays in triage, insufficient physiological monitoring, and underuse of diagnostics are often due to systemic resource constraints and cognitive biases in clinical decision making [79].

Despite these limitations, promising solutions exist. Studies demonstrate that PCT-guided protocols can reduce antibiotic duration by 1.23 to 4.19 days, and infectious disease consultations can shorten treatment by 1 to 1.5 days [29,82,83,84]. Implementation of opt-out stewardship protocols has been associated with a 32% reduction in unnecessary therapy continuation, while computer-assisted decision tools have increased antibiotic-free days from 30% to 42% [85,86,87,88,89]. Nonetheless, these approaches remain underutilized, highlighting the urgent need for standardized, real-time stewardship and decision support integration in clinical practice.

### 3.4. Antibiotic Optimization Through AI: Dosing and De-Escalation Strategies

The optimization of antibiotic therapy is a cornerstone of effective sepsis management. Recent advancements in AI and ML offer data-driven solutions to refine antibiotic prescribing practices, especially in the ICU, where time-sensitive decisions are critical. In recent years, AI and ML technologies have significantly advanced our ability to personalize, de-escalate, and refine antimicrobial regimens, particularly in high-stakes environments like the ICU. These tools support clinicians by providing data-driven insights into dosing, resistance prediction, and individualized treatment decisions.

One of the most prominent initiatives in this field is the KI.SEP study (Künstliche Intelligenz zur Sepsisbehandlung), a prospective observational study evaluating AI’s role in optimizing antibiotic dosages in septic ICU patients [90]. This system integrates patient-specific data, including renal and hepatic function, weight, infection source, and pharmacokinetics, to generate personalized dosing recommendations. Preliminary data from KI.SEP indicate that AI-assisted dosing may improve therapeutic drug levels compared to 30–40% with conventional drug monitoring methods [90].

In parallel, AI models have demonstrated efficacy in guiding de-escalation decisions. For instance, clinical decision support systems (CDSS), such as KINBIOTICS, may be able to provide real-time analysis of microbiological data, resistance trends, and patient trajectories to recommend narrowing or discontinuation of antibiotics [25]. These systems aim to reduce both toxicity risks (e.g., vancomycin-induced nephrotoxicity, which affects 14–28% of patients) and broad-spectrum overuse, which is associated with increased risk of Clostridium difficile infections [25,75,76,77].

A notable cluster-randomized trial investigated the real-world implementation of a machine-learning-assisted sepsis alert (MLASA) system in an emergency department to enhance sepsis care quality [91]. The study was conducted at a tertiary-care hospital emergency department treating over 70,000 patients annually, comparing the MLASA system against conventional human-dedicated sepsis alert processes. Researchers aimed to investigate whether the real-time ML-assisted sepsis alert system could enhance sepsis care quality by increasing the number of ED patients receiving prompt antibiotic treatment and evaluate the diagnostic performance of MLASA compared with traditional sepsis scoring systems (qSOFA, SIRS, MEWS) in early sepsis detection. The study achieved its primary objective, with 68.4% of intervention patients receiving antibiotics within one hour compared to 60.1% of control patients, representing an 8.3 percentage point improvement (OR 1.44; *p* = 0.04). This advantage was even greater for three-hour administration, with 94.5% versus 89.0%, respectively (OR 2.14; *p* = 0.02). The median triage-to-antibiotic time was marginally shorter in the intervention group (46 vs. 50 min), although this was not statistically significant. While the MLASA system successfully expedited treatment, secondary outcomes showed no significant differences in length of stay or 30-day mortality rates between groups. However, the study achieved remarkable validation of the ML model’s diagnostic superiority, with an AUROC of 0.93 compared to traditional tools: qSOFA (0.73), SIRS (0.84), and MEWS (0.86). The ML model demonstrated 79.69% sensitivity, 88.24% specificity, and 98.87% negative predictive value. The study analyzed 574 sepsis patients (256 intervention, 318 control) from 16,579 emergency department visits, providing robust evidence that machine learning can be effectively integrated into clinical workflows to enhance time-sensitive sepsis management. This represents a significant advancement in translating AI technology from theoretical promise to measurable clinical benefit in emergency medicine [91].

Moreover, AI algorithms trained on whole-genome sequencing (WGS) and clinical metadata have shown high accuracy (>90%) in predicting pathogen susceptibility and identifying resistance markers in hours rather than days [92,93]. These tools enable earlier transitions from empiric to targeted therapy, thus shortening the time to effective treatment and improving stewardship outcomes. Table 2 highlights the application of AI in antibiotic guidance across clinical settings. AI-driven dosing tools have improved target drug levels compared to conventional monitoring. Clinical decision support systems have enhanced antimicrobial stewardship by reducing toxicity and minimizing broad-spectrum antibiotic use. Machine learning–assisted alerts in emergency care have increased timely antibiotic administration and demonstrated strong predictive performance.

### 3.5. Resistance Forecasting

The global rise in multidrug-resistant (MDR) and extensively drug-resistant (XDR) pathogens has significantly compromised the efficacy of empiric antibiotic regimens in sepsis. Traditional antimicrobial susceptibility testing (AST) is slow, typically requiring 48–72 h, delaying optimal therapy and facilitating the overuse of broad-spectrum antibiotics. To address this, ML models have emerged as effective tools for forecasting resistance patterns and guiding precision antimicrobial therapy.

A seminal study by Lewin-Epstein et al. demonstrated the potential of ML algorithms to predict antibiotic resistance using only EMR data, including prior antibiotic exposure, comorbidities, and microbiological history [94]. The ensemble model demonstrated strong predictive performance, achieving AUROC scores of 0.73–0.79 when bacterial species information was excluded and substantially improved performance of 0.8–0.88 when bacterial species identity was included. The model’s interpretability and reliance on standard EMR fields make it suitable for real-world clinical deployment.

Further expanding on genomic data integration, Conwill et al. compared ML approaches based on WGS with conventional resistance marker analysis across 107 species/drug combinations relevant to bloodstream infections and found that their ML model (Keynome gAST) achieved 92% median balanced accuracy compared to 80% for resistance-marker-based prediction [92]. Their approach demonstrated superior performance by utilizing the entire bacterial genome rather than limiting analysis to known resistance markers, enabling more accurate antimicrobial susceptibility prediction. These models enable rapid decision making without waiting for phenotypic culture results, accelerating de-escalation and supporting targeted therapy.

Predicting sepsis in maternal units is a specific area of interest. In a study investigating whether bacteremia in pregnant or post-partum women could be predicted by FBC parameters other than the traditional white cell count, it was found that a neutrophil/lymphocyte ratio (NLR) of >20 was the most clinically relevant and interpretable predictor of bacteremia [95]. The ML analysis revealed that all 13 patients with MLR > 20.3 and neutrophils > 19.2 × 10^9^/L had positive blood cultures. The study successfully demonstrated that machine learning approaches could identify more nuanced FBC parameters than traditional white cell counts for bacteremia prediction in the maternity population, providing clinicians with a readily available tool for rapid risk stratification [95].

Web-based tools are increasingly accessible for broader clinical use. For example, the surface-enhanced Raman spectroscopy (SERS)-ATB database, employing deep learning and spectral analysis, identified antibiotics with 98.94% accuracy within seconds, highlighting the role of AI in rapid susceptibility testing and global applicability, including low-resource settings [96].

Specific tools developed for different types of sepsis, such as sepsis-induced coagulopathy, or for the prediction of acute kidney injury in critical care sepsis patients have shown great promise, with excellent metrics reported [97,98].

In ICU settings, ML-driven tools have also been developed to stratify risk for MDR infections and to identify patients likely to develop poor outcomes. For instance, Pan et al. built an ML nomogram to predict MDR *Klebsiella pneumoniae* related septic shock, incorporating patient vitals, comorbidities, and microbiological history [99]. Their model achieved high predictive accuracy (AUROC > 0.90) and helped in early triage and antimicrobial adjustment. Similarly, Özdede et al. [100] used five ML algorithms to predict 14- and 30-day mortality in carbapenem-resistant Acinetobacter baumannii BI. For 14-day mortality; Naïve Bayes achieved the best overall performance (AUC = 0.822, specificity = 0.75), while random forest excelled in 30-day mortality prediction (AUC = 0.854, recall = 0.85, F1 = 0.83). The models were validated using stratified ten-fold cross-validation and consistently identified septic shock as the most critical predictor, emphasizing prevention strategies over treatment interventions [100].

Another innovation, CDSS platforms, such as TREAT, have been designed to predict likely pathogens and their resistance based on regional epidemiology and patient-level clinical data, providing tailored empiric antibiotic recommendations in real time [101,102]. This system showed AUCs of 0.70–0.80 for several Gram-negative pathogens, outperforming physician-based decision making in most scenarios. These systems are particularly valuable in settings with high variability in resistance patterns and limited diagnostic turnaround times.

Several studies represent significant advances in ML applications for sepsis prediction and risk stratification, each addressing different aspects of sepsis management with sophisticated computational approaches.

Liu et al. developed the Septic Shock Risk Predictor (SORP), an innovative early warning system that achieved remarkable predictive performance with an AUC of 0.9458 in the test set, providing a median forewarning time of 13 h before septic shock onset [103]. Using only vital signs and rapid arterial blood gas test features, SORP effectively stratified patients into four distinct risk groups with septic shock incidence rates of 88.6%, 34.5%, 2.5%, and 0.3% for high, medium, low, and ultralow risk groups, respectively. The model’s clinical utility was further enhanced by identifying a previously overlooked patient population (NS_HR) who, despite not meeting Sepsis-3 criteria, exhibited similar pathophysiological characteristics to septic shock patients but demonstrated significantly worse survival outcomes [103].

Park et al. focused on early mortality prediction in ER sepsis patients, developing six ML models, including CatBoost [104]. The CatBoost model demonstrated superior performance, with the highest AUC of 0.800, using clinical variables, while XGBoost achieved an AUC of 0.678 when using SOFA components. Through SHAP value analysis, the study identified albumin, lactate, blood urea nitrogen, and the international normalization ratio as the most significant predictive factors, with the research encompassing 5112 patients across 19 hospitals and achieving accuracy ranging from 0.769 to 0.773 for the top-performing models [104].

Specific models used to predict clinical scenarios in hospitalized cellulitis patients have also been developed [105] by comparing ten different algorithms and artificial neural networks. In external validation using international databases, the artificial neural network model achieved the highest performance, with an AUC of 0.830, demonstrating superior robustness compared to traditional logistic regression models when clinical variables were missing. The study included 6695 patients from the MIMIC-IV database for development and 2506 patients from the YiduCloud database for external validation, with the models showing diagnostic accuracies above 0.9 and the ANN model achieving a diagnostic odds ratio of 9.375 [105].

Pan et al. took a unique approach by focusing specifically on SOFA component scores to predict in-hospital mortality, developing machine learning models that identified the renal system score, central nervous system score, and cardiovascular system score as the three most critical predictive features [106]. The logistic regression and Gaussian Naive Bayes models both achieved AUCs of 0.76, with accuracies of 0.851 and 0.844, respectively, significantly outperforming traditional approaches and demonstrating superior generalization through K-fold cross-validation with average AUROCs of 0.757 ± 0.005 and 0.762 ± 0.006. This comprehensive analysis of 23,889 sepsis patients revealed that machine learning approaches considering the differential weighting of organ system dysfunction significantly improved predictive accuracy compared to simple SOFAs [106].

Moreover, AI tools have extended to fungal infections and *Clostridium difficile* outcomes. AI is increasingly applied to predict antibiotic resistance, with diverse models leveraging clinical, genomic, and spectroscopic data to support rapid and accurate decision-making (Table 3). These systems demonstrate strong predictive performance across varied pathogens and settings, with some models exceeding AUROC values of 0.90 and providing near-instant results. For instance, Ripoli et al. used random forest algorithms to predict candidemia in internal medicine wards (AUROC 0.874), while another study developed an ML model that predicted CDI severity and 60-day recurrence, surpassing traditional severity models in performance (AUROC 0.837) [107,108].

### 3.6. Barriers to Clinical Adoption of AI in Sepsis Care: Challenges in Data Integration, Interpretability, and Ethical Implementation

In addition to describing the range of AI models in recent sepsis research, we critically compared their methodological frameworks, sources of training data, overfitting risks, and ethical considerations. The AI models varied considerably in algorithmic approach, with some using traditional machine learning techniques (such as random forests and logistic regression) and others leveraging more complex deep learning architectures. Notably, models trained on large, multicenter datasets (e.g., MIMIC-III or eICU) demonstrated greater generalizability, while those developed from smaller or single-center cohorts were more susceptible to overfitting and limited external validity. However, explicit reporting of overfitting mitigation, such as cross-validation, regularization, or independent test sets, was inconsistent, and several studies lacked robust external validation, limiting confidence in their real-world applicability.

A central technical challenge remains the reliance on large volumes of high-quality, structured data from diverse sources, including EMRs, laboratory and radiology systems, and bedside monitors. Heterogeneity in data formats, missing values, and lack of standardization across healthcare institutions present significant barriers to model development and deployment, especially in low- and middle-income countries where real-time data integration is often lacking. These factors collectively hinder the scalability and reliability of AI-driven clinical tools.

Interpretability is another major barrier, as many high-performing algorithms—especially deep learning models—function as “black boxes,” making it difficult for clinicians to understand or trust their predictions. Although a minority of studies have incorporated explainable AI (XAI) methods, such as SHAP values or feature importance rankings, widespread adoption remains limited. Clinician trust is more readily gained when AI recommendations are accompanied by clear, clinically plausible explanations.

Ethical considerations, including data privacy, algorithmic bias, and fairness, are also insufficiently addressed in the literature. Models trained on narrow or non-diverse populations risk exacerbating health disparities, and many studies did not report potential demographic performance gaps or fairness evaluations. In addition, robust governance frameworks for privacy, accountability, and transparency are needed to support safe deployment. The lack of clear legal and regulatory guidance, combined with suboptimal integration into clinical workflows, may further contribute to clinician hesitation, alarm fatigue, and inconsistent use of AI tools in practice.

Moreover, although several studies report improved process measures (such as timely antibiotic delivery and reduced inappropriate antibiotic use), robust reductions in mortality, ICU, or hospital length of stay attributable to AI systems remain inconsistently demonstrated. For example, in a cluster-randomized trial, the MLASA system increased prompt antibiotic administration but did not significantly affect 30-day mortality or length of stay. These findings highlight the need for further large-scale, prospective trials with clinical outcomes as primary endpoints.

Overall, while the literature demonstrates significant advances in AI-driven sepsis prediction, important challenges remain regarding methodological rigor, transparency, data integration, and ethical governance. Addressing these gaps will be critical to achieving safe, effective, and equitable clinical translation of AI technologies in sepsis care.

## 4. Future Directions and Recommendations

The integration of artificial intelligence (AI) and machine learning (ML) into sepsis care and antimicrobial stewardship represents a transformative advancement in modern medicine, delivering impressive predictive accuracy with reported AUROC values ranging from 0.68 to 0.99 and prediction windows up to 12 h before clinical recognition [41,90,109]. Nevertheless, widespread clinical adoption remains limited, mainly due to the restricted generalizability of current models, most of which are developed and validated on retrospective, homogeneous, or single-center datasets, thus limiting their utility across diverse patient populations and healthcare environments [21,43,109]. Future research must address this by prioritizing large-scale, prospective, multicenter validation studies to ensure robustness, adaptability, and fairness.

Advances in integrating electronic medical records, biomarkers, rapid genomic diagnostics, and metabolomic data have enhanced predictive performance, such as increasing AUROC from 0.75 to 0.81 [42,56]. However, the real-time use of these continuous data streams within clinical workflows is still uncommon. The next generation of AI solutions should focus on seamless, real-time data integration from wearable biosensors, bedside monitors, and laboratory systems to facilitate dynamic risk assessment, early alerts, and personalized care pathways [110,111,112,113]. Yet, many current AI models lack actionable interpretability and are not designed for straightforward integration with clinical decision support systems, which limits clinician trust and practical deployment.

Despite recent advances, critical research gaps persist. There is a need for rigorous multicenter validation, real-time and interoperable data integration, greater emphasis on explainable AI frameworks for clinical acceptance, and systematic assessments of algorithmic bias and equity. Platforms like TREAT have demonstrated the clinical value of predictive microbiology in empirical therapy guidance [101,102], but adoption will depend on improved interpretability (such as through SHAP values) and full interoperability with hospital information systems [20,25,26]. Furthermore, AI-driven tools should support adaptive therapeutic strategies by incorporating genomic resistance markers, cytokine profiles, and microbiota data, enabling real-time antibiotic personalization and stewardship to counteract resistance [25,90,92,93,99,114].

Streamlined web-based models using minimal variable sets have shown performance comparable to full models [96,108], supporting scalable, low-complexity deployment in resource-limited settings [101,107,115]. As AI tools become more influential in clinical decision making, ethical, regulatory, and legal considerations—including robust data privacy, minimization of algorithmic bias, and transparent recommendations—must be prioritized. The creation of clear regulatory frameworks, combined with clinician oversight and rigorous validation protocols, is essential to ensure that AI augments, rather than replaces, clinical expertise [26,116].

## 5. Conclusions

Our critical review highlights significant heterogeneity among published AI models for sepsis prediction and management. While the majority report high accuracy, substantial variation exists in methodological rigor, transparency, and attention to ethical considerations. Addressing these gaps through standardized reporting, multicenter validation, and adoption of explainable AI techniques will be essential to support the safe and effective clinical integration of AI into sepsis care.

The integration of AI, EMR, biomarkers, and genomic diagnostics heralds a new paradigm for early sepsis detection, real-time monitoring, and personalized treatment. Evidence increasingly supports the clinical value of AI in predicting sepsis onset, optimizing antimicrobial regimens, and identifying multidrug-resistant organisms. Current predictive models have consistently outperformed traditional scoring systems and empirical treatment approaches in sensitivity, specificity, and decision support. However, despite these advances, many AI tools remain confined to research settings, with limited translation into routine clinical practice.

Future efforts must prioritize robust external validation, seamless real-time integration, and user-centered design to fully realize the potential of AI in improving patient outcomes. It is also imperative to ensure equitable access and maintain transparency in AI-supported care, thereby minimizing the risk of perpetuating health disparities.

In summary, AI and machine learning technologies have the capacity to reshape sepsis and infection management by enhancing diagnostic precision, enabling personalized therapies, and contributing to the reduction of antimicrobial resistance. Moving forward, multidisciplinary collaboration among clinicians, data scientists, regulatory bodies, and healthcare institutions will be crucial to responsibly advance the role of AI in medicine and translate these innovations into measurable clinical benefits.

## Figures and Tables

**Table 1:** Comparison of leading AI models for early sepsis prediction: accuracy, data needs, and clinical applicability.

Study/Year	Model Type	Dataset Source	Sample Size	Validation Strategy	Overfitting Mitigation	Interpretability/Ethics	Performance
Li et al., 2020 (TASP) [33]	LightGBM, time-phased ML	3 US hospitals, retrospective EHR	Not specified	5-fold CV, internal/external	Time-phased strategy, cross-validation	Improved interpretability via time-phased cutoffs; focus on early prediction	AUROC 0.845 (internal), utility score 0.430 (internal)
DeepAISE (2020) [34]	Deep learning (time-phased)	Multicenter EHR, ICU	Not specified	Multicenter validation	Implied, not reported	Not specified (focus on accuracy and robustness)	AUROC >0.90
InSight (2016) [35]	Machine learning (vital signs)	Single-center EHR	Not specified	External validation	Minimal feature set, cross-validation	Not interpretable, not reported	AUROC 0.88 (onset), 0.74 (4 h prior); APR 0.59 (onset), 0.27 (4 h prior)
VC-SEPS (2022) [36]	Deep learning	S. Korean hospital, real-world EMR	Not specified	Prospective, external	Not reported	Outperformed SOFA/qSOFA; robust real-world validation	AUROC 0.88, accuracy 87.3%, NPV 0.997, prediction 68 min before diagnosis
Zhao et al., 2022 [37]	Gradient boosting, ML	ICU SA-AKI, multi-institutional	Not specified	Split dev/test cohort	Not specified	SHAP analysis for feature importance, web-based app	AUROC 0.794, accuracy 78.3%, Sens 94.2%, Spec 32.1%
Cimenti et al., 2023 [38]	Extreme gradient boosting	6 Italian hospitals, CBC data	5344	Internal and external	Cross-validation, cautious classification	Excellent performance, focus on blood count/MDW	AUC 0.91–0.98 (internal), 0.75–0.95 (external)
NAVOY (2022) [39]	ML (not specified)	Multicenter ICU, RCT validation	Not specified	Prospective RCT	Not reported	Largest RCT, clinical validation vs. Sepsis-3	AUROC 0.80, Sens 0.80, Spec 0.78, accuracy 0.79
Shi et al., 2022 [40]	Gradient boosting, ML	MIMIC-IV (US), Chinese EHR	Not specified	Internal and external	Not reported	SHAP for global/individual interpretability	AUC > 0.8, accuracy 78% (internal), 63–77% (external)
Nemati et al., 2018 [41]	AISE Algorithm	MIMIC-III, eICU	Not specified	External validation	Regularization, cross-validation	Interpretable predictions, feature highlighting	AUROC 0.83–0.85, Spec/Acc 0.63–0.67, up to 12 h pre-onset
Taneja et al., 2017 [42]	Ensemble ML + biomarkers	Single-center EHR + biomarkers	Not specified	Cross-validation	Cross-validation	Not reported	AUROC 0.75–0.81
Fleuren et al., 2020 [43]	Meta-analysis (various ML/DL)	International pooled, mixed datasets	Not specified	Meta-analytic, multicenter	Mixed, varies by model	Summarizes interpretability and fairness across models	AUROC 0.68–0.99 (varies by model and setting)

Abbreviations: ML, machine learning; DL, deep learning; RNN, recurrent neural network; EHR, electronic health record; ICU, Intensive Care Unit; CBC, complete blood count; MDW, monocyte distribution width; AUROC, Area Under the Receiver Operating Characteristic Curve; NPV, negative predictive value; RCT, randomized controlled trial.

**Table 2:** AI for antibiotic guidance.

Study/Year	Model Type	Dataset Source	Sample Size	Validation Strategy	Overfitting Mitigation	Interpretability/Ethics	Performance/Outcomes
KINBIOTICS [25]	Clinical decision support system (CDSS)	Multicenter microbiology, resistance, clinical data	Not specified	Real-time pilot	Not specified	Supports de-escalation and antimicrobial stewardship	Reduces toxicity (e.g., vancomycin nephrotoxicity) and broad-spectrum overuse
KI.SEP (2023) [90]	Personalized dosing recommender (AI/ML)	ICU patients with sepsis (prospective observational, Germany)	Not specified	Prospective, real-world	Not specified	Transparent dosing logic; focus on safety	AI-assisted dosing achieved optimal drug levels in >50% vs. 30–40% with conventional monitoring
MLASA (2023, cluster RCT) [91]	Machine learning–assisted sepsis alert	ED patients, tertiary hospital (>16,500 visits)	574 sepsis patients	Cluster-randomized trial (intervention vs. control)	Not specified	Integrated into clinical workflow; validated against ethics board	68.4% vs. 60.1% received antibiotics in 1 h; AUROC 0.93; sensitivity 79.7%, specificity 88.2%, NPV 98.9%

Abbreviations: ED: emergency department; ICU: Intensive Care Unit; ML: machine learning; AUROC: Area Under the Receiver Operating Characteristic Curve; CDSS: clinical decision support system; NPV: negative predictive value; RCT: randomized controlled trial.

**Table 3:** AI for antibiotic resistance prediction.

Study/Year	Model Type	Dataset Source/Sample Size	Validation Strategy	Overfitting Mitigation	Interpretability/Ethics	Performance/Outcomes
Conwill et al. [92]	ML (WGS-based, Keynome gAST)	WGS + phenotype, 107 species/drug combinations	Internal split + cross-validation	Not detailed	Genomic explainability; bias addressed	Median balanced accuracy 92% vs. 80% (markers)
Lewin-Epstein et al. [94]	Ensemble ML	EMR data, antibiotic history, comorbidities, microbiology/large Israeli hospital	Internal/external split sample	Not detailed	Feature importance analysis; EMR transparency	AUROC 0.73–0.79 (no species); 0.8–0.88 (w/species)
Maternal sepsis study [95]	ML (FBC parameters)	Pregnant/postpartum, blood cultures, *n* = 13 (MLR > 20.3)	Retrospective cohort	Not specified	Focus on explainable clinical features	100% NLR > 20.3 and neutrophil > 19.2 → bacteremia
SERS-ATB [96]	Deep learning, Raman spectra	SERS-ATB database, multicenter, spectra, *n* not stated	External, real-world, multicenter	Not specified	Global database, accessibility	98.9% accuracy, results in seconds
Pan et al. [99]	ML nomogram	MDR *Klebsiella pneumoniae* septic shock/ICU, China, *n* not stated	Not detailed	Not specified	Model nomogram presented	AUROC > 0.90
Özdede et al. [100]	Five ML algorithms	Carbapenem-resistant *Acinetobacter baumannii* BI, *n* not stated	Stratified 10-fold cross-validation	Not specified	Critical predictor analysis	NB: AUC 0.822 (14d), RF: AUC 0.854 (30d)
TREAT CDSS [101,102]	Clinical decision support	Regional hospital, Gram-negative pathogens	Internal, simulated	Not specified	Recommender logic, scenario analysis	AUC 0.70–0.80, outperforms physicians
Liu et al. (SORP) [103]	Early warning ML system	ICU, vital/lab data, *n* not stated	Internal + test set	Not specified	Forewarning time stratification, risk group stratification	AUC 0.946, 13 h median warning time
Park et al. (ER mortality) [104]	CatBoost, XGBoost, etc.	ER sepsis, 19 Korean hospitals, *n* = 5112	Internal + external, multi-site	Not specified	SHAP, variable importance	CatBoost: AUC 0.800; XGB: AUC 0.678; Acc 0.769–0.773
Cellulitis ANN study [105]	ANN, 10 ML algorithms	MIMIC-IV (*n* = 6695), Yidu-Cloud (*n* = 2506, ext. val.)	External, international	Not specified	Robust to missing data, compared to LR	ANN: AUC 0.830, odds ratio 9.375, Acc > 0.9
Pan et al. (SOFA scores) [106]	LR, Gaussian NB	23,889 sepsis patients, China	K-fold cross-validation	Not specified	Differential weighting, organ-specific explainability	LR, GNB: AUC 0.76, Acc. 0.851–0.844
Ripoli et al. (Candida) [107]	Random forest	Internal medicine wards, candidemia, *n* not stated	Not stated	Not specified	Not specified	AUROC 0.874

Abbreviations: EMR: electronic medical record; ML: machine learning; WGS: whole-genome sequencing; NB: Naive Bayes; RF: random forest; ANN: artificial neural network; LR: logistic regression; SHAP: SHapley Additive Explanations; SERS-ATB: Surface-Enhanced Raman Spectroscopy Antibiotic Database; Acc: accuracy; AUC: area under ROC; AUROC: area under receiver operating characteristic curve; CDI: *Clostridioides difficile* infection; FBC: full blood count.

## Supplementary Materials

The following supporting information can be downloaded at https://www.mdpi.com/article/10.3390/diagnostics15151890/s1, Table S1: QUADAS-2 Assessment.

## References

[1] Cassini A., Allegranzi B., Fleischmann-Struzek C., Kortz T., Markwart R., Saito H., Bonet M., Brizuela V., Mehrtash H., Mingard Ö.T. (2020). Global Report on the Epidemiology and Burden on Sepsis: Current Evidence, Identifying Gaps and Future Directions.

[2] (2020). Global burden of disease and sepsis. Arch. Dis. Child..

[3] Dantes R.B., Kaur H., Bouwkamp B.A., Haass K.A., Patel P., Dudeck M.A., Srinivasan A., Magill S.S., Wilson W.W., Whitaker M. (2023). Sepsis Program Activities in Acute Care Hospitals—National Healthcare Safety Network, United States, 2022. MMWR Morb. Mortal. Wkly. Rep..

[4] Via L.L., Sangiorgio G., Stefani S., Marino A., Nunnari G., Cocuzza S., La Mantia I., Cacopardo B., Stracquadanio S., Spampinato S. (2024). The Global Burden of Sepsis and Septic Shock. Epidemiologia.

[5] Ellaithy I., Elshiekh H., Elshennawy S., Elshenawy S., Al-Shaikh B., Ellaithy A. (2025). Sepsis as a cause of death among elderly cancer patients: An updated SEER database analysis 2000–2021. Ann. Med. Surg..

[6] Martín S., Pérez A., Aldecoa C. (2017). Sepsis and immunosenescence in the elderly patient: A review. Front. Med..

[7] Andersson M., Östholm-Balkhed Å., Fredrikson M., Holmbom M., Hällgren A., Berg S., Hanberger H. (2019). Delay of appropriate antibiotic treatment is associated with high mortality in patients with community-onset sepsis in a Swedish setting. Eur. J. Clin. Microbiol. Infect. Dis..

[8] King J., Chenoweth C.E., England P.C., Heiler A., Kenes M.T., Raghavendran K., Wood W., Zhou S., Mack M., Wesorick D. (2023). Early Recognition and Initial Management of Sepsis in Adult Patients. Ann Arbor (MI): Michigan Medicine University of Michigan. https://www.ncbi.nlm.nih.gov/books/NBK598311/.

[9] Jones Stephen L., Ashton C.M., Kiehne L., Gigliotti E., Bell-Gordon C., Disbot M., Masud F., Shirkey B.A., Wray N.P. (2015). Reductions in Sepsis Mortality and Costs After Design and Implementation of a Nurse-Based Early Recognition and Response Program. Jt. Comm. J. Qual. Patient Saf..

[10] Kumar A., Roberts D., Wood K.E., Light B., Parrillo J.E., Sharma S., Suppes R., Feinstein D., Zanotti S., Taiberg L. (2006). Duration of hypotension before initiation of effective antimicrobial therapy is the critical determinant of survival in human septic shock. Crit. Care Med..

[11] Toker A.K., Kose S., Turken M. (2021). Comparison of sofa score, sirs, qsofa, and qsofa + l criteria in the diagnosis and prognosis of sepsis. Eur. J. Med..

[12] Li Y., Yan C., Gan Z., Xi X., Tan Z., Li J., Li G. (2020). Prognostic values of SOFA score, qSOFA score, and LODS score for patients with sepsis. Ann. Palliat. Med..

[13] Raith E.P., Udy A.A., Bailey M., McGloughlin S., MacIsaac C., Bellomo R., Pilcher D.V., Australian and New Zealand Intensive Care Society (ANZICS) Centre for Outcomes and Resource Evaluation (CORE) (2017). Prognostic Accuracy of the SOFA Score, SIRS Criteria, and qSOFA Score for In-Hospital Mortality Among Adults With Suspected Infection Admitted to the Intensive Care Unit. JAMA.

[14] Shukla P., Rao G.M., Pandey G., Sharma S., Mittapelly N., Shegokar R., Mishra P.R. (2014). Therapeutic interventions in sepsis: Current and anticipated pharmacological agents. Br. J. Pharmacol..

[15] Polat G., Ugan R.A., Cadirci E., Halici Z. (2017). Sepsis and Septic Shock: Current Treatment Strategies and New Approaches. Eur. J. Med..

[16] Vincent J.L., van der Poll T., Marshall J.C. (2022). The End of “One Size Fits All” Sepsis Therapies: Toward an Individualized Approach. Biomedicines.

[17] Ferrari D., Arina P., Edgeworth J., Curcin V., Guidetti V., Mandreoli F., Wang Y., Chaurasia A. (2024). Using interpretable machine learning to predict bloodstream infection and antimicrobial resistance in patients admitted to ICU: Early alert predictors based on EHR data to guide antimicrobial stewardship. PLoS Digit. Health.

[18] Jarczak D., Kluge S., Nierhaus A. (2021). Sepsis—Pathophysiology and Therapeutic Concepts. Front. Med..

[19] Wendland P., Schenkel-Häger C., Wenningmann I., Kschischo M. (2024). An optimal antibiotic selection framework for Sepsis patients using Artificial Intelligence. NPJ Digit. Med..

[20] Cavallaro M., Moran E., Collyer B., McCarthy N.D., Green C., Keeling M.J. (2023). Informing antimicrobial stewardship with explainable AI. PLoS Digit. Health.

[21] Haas R., McGill S.C. (2022). Artificial Intelligence for the Prediction of Sepsis in Adults: CADTH Horizon Scan. Ottawa (ON): Canadian Agency for Drugs and Technologies in Health. https://www.ncbi.nlm.nih.gov/books/NBK596676/.

[22] Huat G.K., Wang L., Yeow A.Y.K., Poh H., Li K., Yeow J.J.L., Tan G.Y.H. (2021). Artificial intelligence in sepsis early prediction and diagnosis using unstructured data in healthcare. Nat. Commun..

[23] Goldschmidt E., Rannon E., Bernstein D., Wasserman A., Roimi M., Shrot A., Coster D., Shamir R. (2025). Predicting appropriateness of antibiotic treatment among ICU patients with hospital-acquired infection. NPJ Digit. Med..

[24] Robert G., Forbes D., Boyer N. (2020). Sepsis: Diagnosis and Management. Am. Fam. Physician.

[25] Düvel J.A., Lampe D., Kirchner M., Elkenkamp S., Cimiano P., Düsing C., Marchi H., Schmiegel S., Fuchs C., Claßen S. (2025). An AI-Based Clinical Decision Support System for Antibiotic Therapy in Sepsis (KINBIOTICS): Use Case Analysis. JMIR Hum. Factors.

[26] Marwaha J.S., Landman A.B., Brat G.A., Dunn T., Gordon W.J. (2022). Deploying digital health tools within large, complex health systems: Key considerations for adoption and implementation. NPJ Digit. Med..

[27] Pepper D.J., Sun J., Rhee C., Welsh J., Powers J.H., Danner R.L., Kadri S.S. (2019). Procalcitonin-Guided Antibiotic Discontinuation and Mortality in Critically Ill Adults: A Systematic Review and Meta-analysis. Chest.

[28] Nobre V., Harbarth S., Graf J.D., Rohner P., Pugin J. (2012). Use of Procalcitonin to Shorten Antibiotic Treatment Duration in Septic Patients. Am. J. Respir. Crit. Care Med..

[29] Kopterides P., Siempos I.I., Tsangaris I., Tsantes A., Armaganidis A. (2010). Procalcitonin-guided algorithms of antibiotic therapy in the intensive care unit: A systematic review and meta-analysis of randomized controlled trials. Crit. Care Med..

[30] Rhee C., Murphy M.V., Li L., Platt R., Klompas M. (2015). Lactate Testing in Suspected Sepsis: Trends and Predictors of Failure to Measure Levels. Crit. Care Med..

[31] Yang J., Hao S., Huang J., Chen T., Liu R., Zhang P., Feng M., He Y., Xiao W., Hong Y. (2023). The application of artificial intelligence in the management of sepsis. Med. Rev..

[32] Yang M.B., Liu C., Wang X., Li Y., Gao H.B., Liu X., Li J. (2020). An Explainable Artificial Intelligence Predictor for Early Detection of Sepsis. Crit. Care Med..

[33] Li X., Xu X., Xie F., Xu X.M., Sun Y.M., Liu X.M., Jia X.B., Kang Y.M., Xie L., Wang F. (2020). A Time-Phased Machine Learning Model for Real-Time Prediction of Sepsis in Critical Care. Crit. Care Med..

[34] Shashikumar S.P., Josef C.S., Sharma A., Nemati S. (2021). DeepAISE—An interpretable and recurrent neural survival model for early prediction of sepsis. Artif. Intell. Med..

[35] Desautels T., Calvert J., Hoffman J., Jay M., Kerem Y., Shieh L., Shimabukuro D., Chettipally U., Feldman M.D., Barton C. (2016). Prediction of Sepsis in the Intensive Care Unit With Minimal Electronic Health Record Data: A Machine Learning Approach. JMIR Med. Inform..

[36] Kim J.-H., Lee K., Kim K.J., Ha E.Y., Kim I.-C., Park S.H., Cho C.-H., Yu G.I., Ahn B.E., Jeong Y. (2025). Validation of an artificial intelligence-based algorithm for predictive performance and risk stratification of sepsis using real-world data from hospitalised patients: A prospective observational study. BMJ Health Care Inf..

[37] Zhao C.-C., Nan Z.-H., Li B., Yin Y.-L., Zhang K., Liu L.-X., Hu Z.-J. (2025). Development and validation of a novel risk-predicted model for early sepsis-associated acute kidney injury in critically ill patients: A retrospective cohort study. BMJ Open.

[38] Campagner A., Agnello L., Carobene A., Padoan A., Del Ben F., Locatelli M., Plebani M., Ognibene A., Lorubbio M., De Vecchi E. (2025). Complete Blood Count and Monocyte Distribution Width–Based Machine Learning Algorithms for Sepsis Detection: Multicentric Development and External Validation Study. J. Med. Internet Res..

[39] Persson I., Macura A., Becedas D., Sjövall F. (2023). Early prediction of sepsis in intensive care patients using the machine learning algorithm NAVOY^®^ Sepsis, a prospective randomized clinical validation study. J. Crit. Care.

[40] Shi S., Zhang L., Zhang S., Shi J., Hong D., Wu S., Pan X., Lin W. (2025). Developing a rapid screening tool for high-risk ICU patients of sepsis: Integrating electronic medical records with machine learning methods for mortality prediction in hospitalized patients—Model establishment, internal and external validation, and visualization. J. Transl. Med..

[41] Nemati S., Holder A., Razmi F., Stanley M.D., Clifford G.D., Buchman T.G. (2018). An Interpretable Machine Learning Model for Accurate Prediction of Sepsis in the ICU. Crit. Care Med..

[42] Taneja I., Reddy B., Damhorst G., Zhao S.D., Hassan U., Price Z., Jensen T., Ghonge T., Patel M., Wachspress S. (2017). Combining Biomarkers with EMR Data to Identify Patients in Different Phases of Sepsis. Sci. Rep..

[43] Fleuren L.M., Klausch T.L.T., Zwager C.L., Schoonmade L.J., Guo T., Roggeveen L.F., Swart E.L., Girbes A.R.J., Thoral P., Ercole A. (2020). Machine learning for the prediction of sepsis: A systematic review and meta-analysis of diagnostic test accuracy. Intensive Care Med..

[44] Zhang G., Shao F., Yuan W., Wu J., Qi X., Gao J., Shao R., Tang Z., Wang T. (2024). Predicting sepsis in-hospital mortality with machine learning: A multi-center study using clinical and inflammatory biomarkers. Eur. J. Med. Res..

[45] Michael M., Bennett N., Plečko D., Horn M., Rieck B., Meinshausen N., Bühlmann P., Borgwardt K. (2023). Predicting sepsis using deep learning across international sites: A retrospective development and validation study. EClinicalMedicine.

[46] Ishan T., Damhorst G.L., Lopez‐Espina C., Zhao S.D., Zhu R., Khan S., White K., Kumar J., Vincent A., Yeh L. (2021). Diagnostic and prognostic capabilities of a biomarker and EMR-based machine learning algorithm for sepsis. Clin. Transl. Sci..

[47] Shimabukuro D.W., Barton C.W., Feldman M.D., Mataraso S.J., Das R. (2017). Effect of a machine learning-based severe sepsis prediction algorithm on patient survival and hospital length of stay: A randomised clinical trial. BMJ Open Respir. Res..

[48] Zheng L., Lin F., Zhu C., Liu G., Wu X., Wu Z., Zheng J., Xia H., Cai Y., Liang H. (2020). Machine Learning Algorithms Identify Pathogen-Specific Biomarkers of Clinical and Metabolomic Characteristics in Septic Patients with Bacterial Infections. Biomed. Res. Int..

[49] Wernly B., Mamandipoor B., Baldia P., Jung C., Osmani V. (2021). Machine learning predicts mortality in septic patients using only routinely available ABG variables: A multi-centre evaluation. Int. J. Med. Inform..

[50] Pappada S.M., Owais M.H., Feeney J.J., Salinas J., Chaney B., Duggan J., Sparkle T., Aouthmany S., Hinch B., Papadimos T.J. (2024). Development and validation of a sepsis risk index supporting early identification of ICU-acquired sepsis: An observational study. Anaesth. Crit. Care Pain. Med..

[51] Sun B., Lei M., Wang L., Wang X., Li X., Mao Z., Kang H., Liu H., Sun S., Zhou F. (2025). Prediction of sepsis among patients with major trauma using artificial intelligence: A multicenter validated cohort study. Int. J. Surg..

[52] Li K., Shi Q., Liu S., Xie Y., Liu J. (2021). Predicting in-hospital mortality in ICU patients with sepsis using gradient boosting decision tree. Medicine.

[53] Li J., Xi F., Yu W., Sun C., Wang X. (2023). Real-Time Prediction of Sepsis in Critical Trauma Patients: Machine Learning–Based Modeling Study. JMIR Form Res..

[54] Boussina A., Shashikumar S.P., Malhotra A., Owens R.L., El-Kareh R., Longhurst C.A., Quintero K., Donahue A., Chan T.C., Nemati S. (2024). Impact of a deep learning sepsis prediction model on quality of care and survival. NPJ Digit. Med..

[55] Sankavi M., Nelson W., Di S., McGillion M., Devereaux P.J., Barr N.G., Petch J. (2021). Machine Learning-Based Early Warning Systems for Clinical Deterioration: Systematic Scoping Review. J. Med. Internet Res..

[56] Langley R.J., Tsalik E.L., van Velkinburgh J.C., Glickman S.W., Rice B.J., Wang C., Chen B., Carin L., Suarez A., Mohney R.P. (2013). Sepsis: An integrated clinico-metabolomic model improves prediction of death in sepsis. Sci. Transl. Med..

[57] Pandey S. (2024). Sepsis, Management & Advances in Metabolomics. Nanotheranostics.

[58] She H., Du Y., Du Y., Tan L., Yang S., Luo X., Li Q., Xiang X., Lu H., Hu Y. (2023). Metabolomics and machine learning approaches for diagnostic and prognostic biomarkers screening in sepsis. BMC Anesthesiol..

[59] D’Onofrio V., Salimans L., Bedenić B., Cartuyvels R., Barišić I., Gyssens I.C. (2020). The Clinical Impact of Rapid Molecular Microbiological Diagnostics for Pathogen and Resistance Gene Identification in Patients With Sepsis: A Systematic Review. Open Forum Infect. Dis..

[60] Eubank T.A., Long S.W., Perez K.K. (2020). Role of Rapid Diagnostics in Diagnosis and Management of Patients with Sepsis. J. Infect. Dis..

[61] Riedel S., Carroll K.C. (2016). Early Identification and Treatment of Pathogens in Sepsis: Molecular Diagnostics and Antibiotic Choice. Clin. Chest Med..

[62] Sinha M., Jupe J., Mack H., Coleman T.P., Lawrence S.M., Fraley S.I. (2018). Emerging technologies for molecular diagnosis of sepsis. Clin. Microbiol. Rev..

[63] Plata-Menchaca E.P., Ferrer R. (2018). Life-support tools for improving performance of the Surviving Sepsis Campaign Hour-1 bundle. Med. Intensiv..

[64] Ranieri V.M., Thompson B.T., Barie P.S., Dhainaut J.-F., Douglas I.S., Finfer S., Gårdlund B., Marshall J.C., Rhodes A., Artigas A. (2012). Drotrecogin alfa (activated) in adults with septic shock. N. Engl. J. Med..

[65] Levy M.M., Evans L.E., Rhodes A. (2018). The Surviving Sepsis Campaign Bundle: 2018 update. Intensive Care Med..

[66] Rhodes A., Evans L.E., Alhazzani W., Levy M.M., Antonelli M., Ferrer R., Kumar A., Sevransky J.E., Sprung C.L., Nunnally M.E. (2017). Surviving Sepsis Campaign: International Guidelines for Management of Sepsis and Septic Shock: 2016. Intensive Care Med..

[67] Martínez M.L., Plata-Menchaca E.P., Ruiz-Rodríguez J.C., Ferrer R. (2020). An approach to antibiotic treatment in patients with sepsis. J. Thorac. Dis..

[68] Zhang J., Shi H., Xia Y., Zhu Z., Zhang Y. (2024). Knowledge, attitudes, and practices among physicians and pharmacists toward antibiotic use in sepsis. Front. Med..

[69] Seok H., Jeon J.H., Park D.W. (2020). Antimicrobial Therapy and Antimicrobial Stewardship in Sepsis. Infect. Chemother..

[70] Donkor E.S., Codjoe F.S. (2019). Methicillin Resistant Staphylococcus aureus and Extended Spectrum Beta-lactamase Producing Enterobacteriaceae: A Therapeutic Challenge in the 21st Century. Open Microbiol. J..

[71] Department of Health Antimicrobial Resistance Strategy Analytical Working Group (2016). Antimicrobial Resistance: Empirical and Statistical Evidence-Base. https://assets.publishing.service.gov.uk/media/5a7f417ce5274a2e87db4be3/AMR_EBO_2016.pdf.

[72] Llor C., Bjerrum L. (2014). Antimicrobial resistance: Risk associated with antibiotic overuse and initiatives to reduce the problem. Ther. Adv. Drug Saf..

[73] Liu V.X., Fielding-Singh V., Greene J.D., Baker J.M., Iwashyna T.J., Bhattacharya J., Escobar G.J. (2017). The timing of early antibiotics and hospital mortality in sepsis. Am. J. Respir. Crit. Care Med..

[74] Ha D.R., Haste N.M., Gluckstein D.P. (2017). The Role of Antibiotic Stewardship in Promoting Appropriate Antibiotic Use. Am. J. Lifestyle Med..

[75] Filippone E.J., Kraft W.K., Farber J.L. (2017). The Nephrotoxicity of Vancomycin. Clin. Pharmacol. Ther..

[76] Wong-Beringer A., Joo J., Tse E., Beringer P. (2011). Vancomycin-associated nephrotoxicity: A critical appraisal of risk with high-dose therapy. Int. J. Antimicrob. Agents.

[77] Song J.H., Kim Y.S. (2019). Recurrent Clostridium difficile Infection: Risk Factors, Treatment, and Prevention. Gut Liver.

[78] Saleem M., Deters B., de la Bastide A., Korzen M. (2019). Antibiotics Overuse and Bacterial Resistance. Ann. Microbiol. Res..

[79] Husabø G., Nilsen R.M., Flaatten H., Solligård E., Frich J.C., Bondevik G.T., Braut G.S., Walshe K., Harthug S., Hovlid E. (2020). Early diagnosis of sepsis in emergency departments, time to treatment, and association with mortality: An observational study. PLoS ONE.

[80] Tang F., Yuan H., Li X., Qiao L. (2024). Effect of delayed antibiotic use on mortality outcomes in patients with sepsis or septic shock: A systematic review and meta-analysis. Int. Immunopharmacol..

[81] Neilson H.K.M., Fortier J.H.M., Finestone P.R., Ogilby C.M.R., Liu R.M., Bridges E.J.M., Garber G.E.M. (2023). Diagnostic Delays in Sepsis: Lessons Learned From a Retrospective Study of Canadian Medico-Legal Claims. Crit Care Explor..

[82] Lesprit P., Landelle C., Brun-Buisson C. (2013). Clinical impact of unsolicited post-prescription antibiotic review in surgical and medical wards: A randomized controlled trial. Clin. Microbiol. Infect..

[83] Kiya G.T., Asefa E.T., Abebe G., Mekonnen Z. (2024). Procalcitonin Guided Antibiotic Stewardship. Biomark. Insights.

[84] Willmon J., Subedi B., Girgis R., Noe M. (2021). Impact of Pharmacist-Directed Simplified Procalcitonin Algorithm on Antibiotic Therapy in Critically Ill Patients With Sepsis. Hosp. Pharm..

[85] Moehring R.W., E Yarrington M., Warren B.G., Lokhnygina Y., Atkinson E., Bankston A., Coluccio J., David M.Z., Davis A., Davis J. (2021). 14. Effects of an Opt-Out Protocol for Antibiotic De-escalation among Selected Patients with Suspected Sepsis: The DETOURS Trial. Open Forum Infect. Dis..

[86] Moehring R.W., E Yarrington M., Warren B.G., Lokhnygina Y., Atkinson E., Bankston A., Collucio J., David M.Z., E Davis A., Davis J. (2023). Evaluation of an Opt-Out Protocol for Antibiotic De-Escalation in Patients With Suspected Sepsis: A Multicenter, Randomized, Controlled Trial. Clin. Infect. Dis..

[87] Nachtigall I., Tafelski S., Deja M., Halle E., Grebe M.C., Tamarkin A., Rothbart A., Uhrig A., Meyer E., Musial-Bright L. (2014). Long-term effect of computer-assisted decision support for antibiotic treatment in critically ill patients: A prospective ‘before/after’ cohort study. BMJ Open.

[88] Niederman M.S., Baron R.M., Bouadma L., Calandra T., Daneman N., DeWaele J., Kollef M.H., Lipman J., Nair G.B. (2021). Initial antimicrobial management of sepsis. Crit. Care.

[89] Li F., Wang S., Gao Z., Qing M., Pan S., Liu Y., Hu C. (2024). Harnessing artificial intelligence in sepsis care: Advances in early detection, personalized treatment, and real-time monitoring. Front. Med..

[90] Marko B., Palmowski L., Nowak H., Witowski A., Koos B., Rump K., Bergmann L., Bandow J., Eisenacher M., Günther P. (2024). Employing artificial intelligence for optimising antibiotic dosages in sepsis on intensive care unit: A study protocol for a prospective observational study (KI.SEP). BMJ Open.

[91] Kijpaisalratana N., Saoraya J., Nhuboonkaew P., Vongkulbhisan K., Musikatavorn K. (2024). Real-time machine learning-assisted sepsis alert enhances the timeliness of antibiotic administration and diagnostic accuracy in emergency department patients with sepsis: A cluster-randomized trial. Intern. Emerg. Med..

[92] Conwill A., Sater M., Worley N., Wittenbach J., Huntley M.H. (2023). 966. Large-Scale Evaluation of AST Prediction using Resistance Marker Presence/Absence vs. Machine Learning on WGS Data. Open Forum Infect. Dis..

[93] Jana T., Sarkar D., Ganguli D., Mukherjee S.K., Mandal R.S., Das S. (2024). ABDpred: Prediction of active antimicrobial compounds using supervised machine learning techniques. Indian. J. Med. Res..

[94] Lewin-Epstein O., Baruch S., Hadany L., Stein G.Y., Obolski U. (2021). Predicting Antibiotic Resistance in Hospitalized Patients by Applying Machine Learning to Electronic Medical Records. Clin. Infect. Dis..

[95] Mooney C., Eogan M., Áinle F.N., Cleary B., Gallagher J.J., O’LOughlin J., Drew R.J. (2021). Predicting bacteraemia in maternity patients using full blood count parameters: A supervised machine learning algorithm approach. Int. J. Lab. Hematol..

[96] Yuan Q., Tang J.-W., Chen J., Liao Y.-W., Zhang W.-W., Wen X.-R., Liu X., Chen H.-J., Wang L. (2025). SERS-ATB: A comprehensive database server for antibiotic SERS spectral visualization and deep-learning identification. Environ. Pollut..

[97] Tan R., Ge C., Wang J., Yang Z., Guo H., Yan Y., Du Q. (2025). Interpretable machine learning model for early morbidity risk prediction in patients with sepsis-induced coagulopathy: A multi-center study. Front. Immunol..

[98] Shi J., Han H., Chen S., Liu W., Li Y. (2024). Machine learning for prediction of acute kidney injury in patients diagnosed with sepsis in critical care. PLoS ONE..

[99] Pan S., Shi T., Ji J., Wang K., Jiang K., Yu Y., Li C. (2025). Developing and validating a machine learning model to predict multidrug-resistant Klebsiella pneumoniae-related septic shock. Front. Immunol..

[100] Özdede M., Zarakolu P., Metan G., Eser Ö.K., Selimova C., Kızılkaya C., Elmalı F., Akova M. (2024). Predictive modeling of mortality in carbapenem-resistant Acinetobacter baumannii bloodstream infections using machine learning. J Investig. Med..

[101] Paul M., Nielsen A.D., Goldberg E., Andreassen S., Tacconelli E., Almanasreh N., Frank U., Cauda R., Leibovici L., TREAT Study Group (2007). Prediction of specific pathogens in patients with sepsis: Evaluation of TREAT, a computerized decision support system. J. Antimicrob. Chemother..

[102] Paul M., Andreassen S., Nielsen A.D., Tacconelli E., Almanasreh N., Fraser A., Yahav D., Ram R., Leibovici L., TREAT Study Group (2006). Prediction of bacteremia using TREAT, a computerized decision-support system. Clin. Infect. Dis..

[103] Liu G., Zheng S., He J., Zhang Z.-M., Wu R., Yu Y., Fu H., Han L., Zhu H., Xu Y. (2025). An Easy and Quick Risk-Stratified Early Forewarning Model for Septic Shock in the Intensive Care Unit: Development, Validation, and Interpretation Study. J. Med. Internet Res..

[104] Park S.W., Yeo N.Y., Kang S., Ha T., Kim T.-H., Lee D., Kim D., Choi S., Kim M., Lee D. (2024). Early Prediction of Mortality for Septic Patients Visiting Emergency Room Based on Explainable Machine Learning: A Real-World Multicenter Study. J. Korean Med. Sci..

[105] Chen X., Hu L., Yu R. (2024). Development and external validation of machine learning-based models to predict patients with cellulitis developing sepsis during hospitalisation. BMJ Open..

[106] Pan X., Xie J., Zhang L., Wang X., Zhang S., Zhuang Y., Lin X., Shi S., Shi S., Lin W. (2023). Evaluate prognostic accuracy of SOFA component score for mortality among adults with sepsis by machine learning method. BMC Infect. Dis..

[107] Ripoli A., Sozio E., Sbrana F., Bertolino G., Pallotto C., Cardinali G., Meini S., Pieralli F., Azzini A.M., Concia E. (2020). Personalized machine learning approach to predict candidemia in medical wards. Infection.

[108] Madden G.R., Boone R.H., Lee E., Sifri C.D., Petri W.A. (2024). Predicting Clostridioides difficile infection outcomes with explainable machine learning. EBioMedicine.

[109] Islam K.R., Prithula J., Kumar J., Tan T.L., Reaz M.B.I., Sumon S.I., Chowdhury M.E.H. (2023). Machine Learning-Based Early Prediction of Sepsis Using Electronic Health Records: A Systematic Review. J. Clin. Med..

[110] Sharma V., Ali I., van der Veer S., Martin G., Ainsworth J., Augustine T. (2021). Adoption of clinical risk prediction tools is limited by a lack of integration with electronic health records. BMJ Health Care Inform..

[111] Barton C., Chettipally U., Zhou Y., Jiang Z., Lynn-Palevsky A., Le S., Calvert J., Das R. (2019). Evaluation of a machine learning algorithm for up to 48-hour advance prediction of sepsis using six vital signs. Comput. Biol. Med..

[112] Sadasivuni S., Saha M., Bhatia N., Banerjee I., Sanyal A. (2022). Fusion of fully integrated analog machine learning classifier with electronic medical records for real-time prediction of sepsis onset. Sci. Rep..

[113] Gao W., Pei Y., Liang H., Lv J., Chen J., Zhong W. (2021). Multimodal AI system for the rapid diagnosis and surgical prediction of necrotizing enterocolitis. IEEE Access.

[114] Jacobs L., Wong H.R. (2016). Emerging infection and sepsis biomarkers: Will they change current therapies?. Expert Rev. Anti Infect. Ther..

[115] Sakagianni A., Koufopoulou C., Feretzakis G., Kalles D., Verykios V.S., Myrianthefs P., Fildisis G. (2023). Using Machine Learning to Predict Antimicrobial Resistance-A Literature Review. Antibiotics.

[116] Watkins R.R., Bonomo R.A., Rello J. (2022). Managing sepsis in the era of precision medicine: Challenges and opportunities. Expert Rev. Anti Infect. Ther..

